# Nursing Workload as a Risk Factor for Healthcare Associated Infections in ICU: A Prospective Study

**DOI:** 10.1371/journal.pone.0052342

**Published:** 2012-12-27

**Authors:** Renata M. Daud-Gallotti, Silvia F. Costa, Thais Guimarães, Katia Grillo Padilha, Evelize Naomi Inoue, Tiago Nery Vasconcelos, Fernanda da Silva Cunha Rodrigues, Edizângela Vasconcelos Barbosa, Walquíria Barcelos Figueiredo, Anna S. Levin

**Affiliations:** 1 Humanization Technical Division, Hospital das Clínicas of the University of São Paulo; Faculty of Medicine, Universidade Nove de Julho, São Paulo, Brazil; 2 Department of Infectious Diseases and LIM-54, Faculty of Medicine, University of São Paulo, São Paulo, São Paulo, Brazil; 3 Department of Infection Control, Faculty of Medicine, Hospital das Clínicas of University of São Paulo, São Paulo, São Paulo, Brazil; 4 School of Nursing, University of São Paulo, São Paulo, São Paulo, Brazil; 5 Faculty of Medicine, University of São Paulo, São Paulo, São Paulo, Brazil; 6 Nursing Division, Faculty of Medicine, Hospital das Clínicas of University of São Paulo, São Paulo, São Paulo, Brazil; University of Louisville, United States of America

## Abstract

**Introduction:**

Nurse understaffing is frequently hypothesized as a potential risk factor for healthcare-associated infections (HAI). This study aimed to evaluate the role of nursing workload in the occurrence of HAI, using Nursing Activities Score (NAS).

**Methods:**

This prospective cohort study enrolled all patients admitted to 3 Medical ICUs and one step-down unit during 3 months (2009). Patients were followed-up until HAI, discharge or death. Information was obtained from direct daily observation of medical and nursing rounds, chart review and monitoring of laboratory system. Nursing workload was determined using NAS. Non-compliance to the nurses’ patient care plans (NPC) was identified. Demographic data, clinical severity, invasive procedures, hospital interventions, and the occurrence of other adverse events were also recorded. Patients who developed HAI were compared with those who did not.

**Results:**

195 patients were included and 43 (22%) developed HAI: 16 pneumonia, 12 urinary-tract, 8 bloodstream, 2 surgical site, 2 other respiratory infections and 3 other. Average NAS and average proportion of non compliance with NPC were significantly higher in HAI patients. They were also more likely to suffer other adverse events. Only excessive nursing workload (OR: 11.41; p: 0.019) and severity of patient’s clinical condition (OR: 1.13; p: 0.015) remained as risk factors to HAI.

**Conclusions:**

Excessive nursing workload was the main risk factor for HAI, when evaluated together with other invasive devices except mechanical ventilation. To our knowledge, this study is the first to evaluate prospectively the nursing workload as a potential risk factor for HAI, using NAS.

## Introduction

Invasive procedures have been the subject of studies on the epidemiology of healthcare-associated infection (HAI) and are considered traditional risk factors, such as central venous catheters for bloodstream infections and mechanical ventilation for pneumonias [1], [2]. Part of these infections is caused by exogenous contamination, linked to inadequate practices of sterile technique. Examples of this are unsafe injection practices including, but are not limited to, reuse of syringes for multiple patients or to access shared medications, administration of medication from a single-dose/single-use vial to multiple patients, and failure to use aseptic technique when preparing and administering injections [3]. A recent outbreak clearly illustrates this point [4]. On the other hand when safe practices in injection and medication vial utilization are adopted, infection is extremely rare, especially in the outpatient setting [5].

Extensive studies on prevention of healthcare-associated infections have led to the determination of prevention guidelines [1], [2]. However, if prevention measures are well known, the implementation of these is the current major challenge. Implementation of “bundles” that include a limited number of interventions that are well backed by a high level of scientific evidence, are one of the main strategies of prevention [6]. However individually studied interventions may not be effective when implemented in a bundle [7].

Nurse understaffing is frequently hypothesized as a potential risk factor for HAI [8], but its role has not been completely evaluated [9]. Invasive procedures, besides being a risk factor *per se*, may contribute to the workload of the healthcare workers (HCW), especially to the nursing personnel.

The aim of this study was to evaluate the role of nursing workload in the occurrence of HAI in medical intensive care units, using a specific scoring system (Nursing Activities Score - NAS).

## Methods

### Study Design: Prospective Cohort of Patients Under Intensive Care

The study was conducted at Hospital das Clínicas (HC) in the city of São Paulo, Brazil. Our Institutional Review Board approved the study and waived the need for patient consent forms.

São Paulo is among the largest cities in Latin America, with 11 million inhabitants. HC is a tertiary-care teaching hospital complex affiliated to the University of São Paulo. HC is a government hospital predominantly for patients covered by the publicly funded Brazilian National Health Service. It has approximately 2,000 beds distributed in 7 institutes. The Central Institute (main building) has 894 beds including 100 ICU beds. This study was conducted during the period from May 25, 2009 to August 25, 2009 in 3 Medical ICUs: General Medicine (6 beds), Pneumology (4 beds) and Emergency (8 beds) plus a step-down unit with 9 beds, thus including a total of 27 beds. The average length of patient stay in these units is 7 days. In the ICU 2 beds are assigned to each nursing HCW and in the step-down unit each HCW is assigned 3 beds. During the study period, monthly absenteeism of HCWs varied from 1.6% to 5.7%.

All the patients aged 12 years or more admitted to these units were included in the study. Only the first admission to the unit was considered. The patients were followed-up until a HAI occurred, or until their discharge from the unit or until their death, whichever occurred earlier.

All the medical rounds (once daily every morning) and the nursing rounds that occurred daily at 7 am and at 7 pm were followed by one of the members of the research team. The persons involved in data collection and observation did not belong to the staff of the units and received training during one-month previous to the study.

The following information was obtained from the rounds, the patients’ records and from the computerized laboratory system:

—Demographic data from the patients: sex, age, origin (emergency room, patient wards, community, other hospitals or institutions).
—Severity of patient condition on admission to the unit using the scores APACHE II score [10]. A daily evaluation of severity was performed using the scores SAPS II [11] and SOFA [12]. For the analysis, an average of each score was calculated for the follow-up period for each patient.
—Length of stay in the hospital and in the unit under study.
—Underlying diseases, including the Charlson Score [13], [14]
—Non-compliance to the nurses’ patient care plans (NPC): The execution of the daily NPC by the nursing team was evaluated by our research group from the review of medical charts and during the direct observation of rounds. The total number of items prescribed in the NPC and the number of those items that were not performed were registered. Non-compliance to NPC was defined as the proportion of items that were not performed by the nursing team among the total prescribe items.
—Evaluation of the nursing workload by using the Nursing Activities Score (NAS) [15]. This score derives from a daily evaluation that takes into account 23 items divided into 7 categories: monitoring and controls, respiratory support, cardiac support, renal support, metabolic support and specific intervention performed inside and outside the ICU. The score estimates the proportion of time that nursing staff is required to spend assisting the patient during a work shift. A NAS form was filled out at the end of each shift for each patient by the nursing professional directly responsible for his or her care. All personnel were previously trained to use NAS. The daily NAS was determined to each patient during the follow up period in order to calculate the average NAS of this period, for each patient. Considering that in the intensive care units 2 beds were assigned to each nursing HCW, we considered that an average NAS ≥51% per patient during the follow-up up indicated an excessive workload.
—The use of invasive procedures during the hospitalization in the units under study, including mechanical ventilation, central venous catheter, peripheral venous catheter, urinary catheter, surgery, drainage, dialysis, endoscopy, bronchoscopy, total parenteral nutrition and enteral nutrition. The total number of days under these procedures was also evaluated.
—Communication failures – during 5 hours each day the practices in the units were observed by members of the research team and the average daily number of failures in communication between healthcare professionals. The following situations were considered communication failures: misunderstood orders; opposite information presented in medical and nursing rounds in the same day, quarrels involving healthcare team including the patient or not. A rate was calculated for each patient by dividing the total number of failures in communication by the number of days of follow-up.
—Use of blood products.
—Proportion of days in which the patient’s bed head was elevated (>30°).

The outcome evaluated was the acquisition of HAI. Data on HAI were obtained by active surveillance performed by the Infection Control nurses using definitions by the Centers for disease Control and Prevention [16]. Infections that occurred within the first 48 hours after discharge from the unit were considered as acquired in the unit.

For the patients who developed a HAI, only the first infection was considered and all the variables were evaluated until the occurrence of this infection. For the patients who did not acquire an infection, the full period of hospitalization in the unit was evaluated.

Adverse events (AEs) that occurred to patients during the entire hospitalization were evaluated: pressure ulcers, episodes of hypoglycemia, phlebitis and falls. The following events were also registered: whether the patient remained in the ICU for more than 24 hours despite medical indication of discharge from the unit, occurrence of consultations by other medical specialists delayed for more than 24 hours, number of cancelations of programmed surgeries and number of cancelations of invasive procedures. All the patients were also evaluated as to death during hospitalization in the unit and in the hospital.

### Data Analysis

A data base was created using Microsoft Office Excel 2007 (Microsoft, Redmond WA, USA) and the statistical analyses were performed using the software Stata version 10.0 (StataCorp, College Station TX).

Patients who developed a HAI were compared with those who did not. Relative risks and 95% confidence intervals were determined for dichotomous variables. Categorical variables were compared using the chi square test. ANOVA was used to compare continuous variables. A p-value <0.05 was considered to be statistically significant. A multivariate analysis was performed to evaluate potential factors associated with acquiring a HAI, using multiple logistic regression. The variables that were statistically significant in the bivariate analysis and those considered by the authors to be biologically plausible were tested.

## Results

During the study period 195 patients were included: 149 (76%) admitted initially to the intensive care units and 46 (24%) to the step-down unit. The patients could be reallocated from one unit to another according their clinical condition.

Forty-three patients (22%) developed HAI: 16 (37%) with pneumonia; 12 (28%) urinary tract infections; 8 (19%) bloodstream infections; 2 surgical site infections, 2 other respiratory infections and 3 other infections (abdominal, soft tissue and vascular, respectively).

The bivariate analysis of the factors associated with acquiring a HAI can be seen in Tables 1 and 2. The severity of patient’s clinical condition evaluated using different scores (APACHE II, SOFA and SAPS II); longer ICU stay; excessive nursing workload; and greater non-compliance with the NPC were all associated with HAI in the bivariate analysis. In addition, the following variables were also associated with acquiring a HAI: use of invasive procedures (mechanical ventilation, urinary catheters, central venous catheters and endoscopy); use of blood products; enteral nutrition; and hemodialysis.

**Table 1:** Bivariate analysis of continuous variables potentially associated with acquiring a healthcare-associated infection (HAI) in 3 intensive care units and one step-down unit in Hospital das Clínicas, University of São Paulo, Brazil (May 2009–August 2009).

Variáveis	Patients who acquiredHAI (n:43)	Patients who did NOTacquire HAI (n:152)	*p*
**Age** (years)-mean(SD)	56.2 (18.5)	50.9 (19.8)	0.12
Median (range)	59 (19–86)	52.5 (15–96)	
**Charlton score** (points)-mean(SD)	2.81 (2.37)	2.26 (2.24)	0.16
Median (range)	2 (0–9)	2 (0–9)	
**APACHE II score** (points)-mean(SD)	19.1 (5.0)	15.4 (7.3)	**0.002**
Median (range)	20 (8–29)	14 (2–48)	
**SAPS II (points**)-mean (SD)	37.0 (9.2)	28.0 (16.1)	**<0.001**
Median (range)	35.8 (16.1–56)	24.8 (1.4–90)	
**SOFA (points)**-mean(SD)	6.23 (2.62)	4.61 (3.66)	**0.007**
Median (range)	6.08 (0.43–13.17)	3.65 (0–18)	
**ICU length of stay** (days)-mean(SD)	11.3 (8.0)	8.3 (7.4)	**0.02**
Median (range)	9 (4–46)	6 (1–62)	
**NAS** (%)-mean(SD)	81.2 (16.2)	66.7 (20.3)	**<0.001**
Median (range)	81.9 (37.8–131.8)	65.5 (28.9–145.5)	
**Daily proportion of non-compliance with the nurses’** **patient care plans (%)**			**<0.001**
Mean (SD)	23.4 (24.5)	14.1 (12.4)	
Median (range)	19.0 (0–153.3)	12.0 (0–43.2)	
**Daily Communication failures** (number)			0.44
Mean (SD)	0.81 (1.23)	0.64 (1.34)	
Median (range)	0 (0–6)	0 (0–7)	
**Proportion of days in which bed not elevated >30°**(%)-mean(SD)	2.3 (15.2)	2.5 (11.4)	0.94
Median (range)	0 (0–100)	0 (0–100)	
**Days of urinary catheter use**-mean(SD)	8.49 (6.07)	3.37 (4.84)	**<0.001**
Median (range)	7 (0–30)	2 (0–30)	
**Days of CVC use**-mean (SD)	8.37 (5.80)	3.26 (6.08)	**<0.001**
Median (range)	7 (0–24)	0.5 (0–59)	
**Days of mechanical ventilation**-mean (SD)	5.53 (5.44)	1.64 (3.02)	**<0.001**
Median (range)	5 (0–20)	0 (0–16)	
**Days of enteral nutrition**-mean(SD)	6.47 (5.88)	1.22 (5.33)	**<0.001**
Median (range)	5 (0–22)	0 (0–49)	
**Mean number of surgeries** (SD)	0.23 (0.57)	0.16 (0.39)	0.37
Median (range)	0 (0–2)	0 (0–2)	
**Days of TPN**-mean(SD)	0.14 (0.91)	0.32 (3.97)	0.77
Median (range)	0 (0–6)	0 (0–49)	

SD: standard deviation; CVC: central venous catheter; TPN: total parenteral nutrition; NAS: nursing Activities Score; ICU: Intensive Care Unit

**Table 2:** Bivariate analysis of categorical variables potentially associated with acquiring a healthcare-associated infection (HAI) in 3 intensive care units and one step-down unit in Hospital das Clínicas, University of São Paulo, Brazil (May 2009–August 2009).

Variables	Patients who acquired HAI (n: 43)	Patients who did NOT acquire HAI (n: 152)	Relative risk	95% confidence interval	p
**Male sex**	23	76	1.12	0.66–1.89	0.69
**Presence of comorbidity**	34	109	1.52	0.75–3.05	0.23
**Origin**					0.93
• Hospital ward	8	31			
• Emergency room	25	89			
• Operating room	4	16			
• Other	6	16			
**Excessive nursing workload (NAS≥51%)**	40	116	**3.33**	**1.09**–**10.21**	**0.016**
**Medical/hospital interventions**					
• Enteral nutrition	33	19	**9.08**	**4.82**–**17.08**	**<0.001**
• CVC	39	76	**6.78**	**2.52**–**18.23**	**<0.001**
• Mechanical ventilation	32	50	**4.01**	**2.15**–**7.48**	**<0.001**
• Urinary catheter	40	91	**6.51**	**2.09**–**20.26**	**<0.001**
• Transfusion	24	32	**3.14**	**1.87**–**5.25**	**<0.001**
• Hemodialysis	14	20	**2.29**	**1.36**–**3.84**	**0.003**
• Endoscopy	8	10	**2.25**	**1.24**–**4.08**	**0.016**
• Total parenteral nutrition	1	1	2.30	0.56–9.42	0.34
• Bronchoscopy	4	11	1.23	0.51–2.98	0.65
• Drains	3	10	1.05	0.38–2.94	0.93
• Surgery	7	24	1.03	0.50–2.10	0.94
**Death during stay in the unit**	17	23	**2.53**	**1.53**–**4.19**	**<0.001**
**Death during hospital stay**	19	29	**2.42**	**1.46**–**4.02**	**<0.001**
**Occurrence of other adverse events during hospitalization**					
• Pressure ulcer	31	28	**5.95**	**3.29**–**10.77**	**<0.001**
• Cancellation of programmed surgery	3	1	**3.58**	**1.91**–**6.72**	**0.01**
• Hypoglicemia	15	27	**1.95**	**1.15**–**3.30**	**0.016**
• Phlebitis	11	26	1.47	0.82–2.63	0.21
• Delay >24 h of consults	8	21	1.31	0.68–2.53	0.44
• Hospitalization >24 h after discharge ordered	10	33	1.07	0.58–1.99	0.83
• Cancellation of invasive procedure	1	9	0.44	0.07–2.88	0.35

CVC: central venous catheter; NAS: Nursing activity score.

Patients who acquired HAI were more likely to suffer other AEs during their stay in the ICU: pressure ulcers, hypoglycemia and death in the unit (Table 2). They were also more at risk to suffer a cancellation of a programmed surgical operation and to die during their entire hospital stay.

The following variables were tested in the multivariate analysis: age (10-year strata), severity of clinical condition (SOFA score); excessive workload (NAS>51%); non-compliance with the nurses’ patient care plans (10% strata); and use of hospital interventions (central venous catheter; use urinary catheter; receipt of red blood cell concentrate; hemodialysis; endoscopy).

Excessive workload was the most important independent risk factor significantly associated with acquiring a HAI among our patients, with OR estimates above 11.0 (OR: 11.41; 95% CI: 1.49–87.28; p: 0.019). The severity of patient’s clinical condition was also significantly associated with HAI, with a lower strength of association (OR: 1.13; 95% CI: 1.02–1.24; p: 0.015). Table 3 shows the multivariate analysis.

**Table 3:** Multivariate analysis evaluating factors associated with acquiring a healthcare associated infection in 3 intensive care units and one step-down unit in Hospital das Clínicas, University of São Paulo, Brazil (May 2009–August 2009).

Variable	Odds ratio	95% confidence interval	*p*
Excessive nursing workload (NAS ≥51%)	11.41	1.49–87.28	0.019
Severity of clinical condition (SOFA score)	1.13	1.02–1.24	0.015

NAS: Nursing Activities Score; SOFA: Sepsis-related Organ Failure Assessment.

It is important to point out that a model including mechanical ventilation, in addition to the other described variables, was also tested. In this situation, only mechanical ventilation remained in the model.

## Discussion

In our prospective study with direct observation of clinical ICUs of an academic tertiary hospital, we found that excessive nursing workload was the most important risk factor for acquiring HAI, followed by severity of clinical condition.

HAI can originate from a number of different factors: exogenous contamination, linked to inadequate practices of sterile technique such as unsafe injection practices [3], and the use of invasive devices, traditionally considered to be risk factors for infections [1], [2]. Knowledge on how to prevent most infections is well established, however all adequate practices and techniques must ultimately be embraced and applied by the heath care workers. This is currently the major challenge.

Most of the studies of risk factors for infection are retrospective and this limits the evaluation of administrative and dynamic functional factors, such as nursing workload, interpersonal communication and programmed procedures, such as surgery and exams. All these situations could be analyzed in our prospective study to some extent. Furthermore, this study is part of a more ample prospective one, focusing on patient safety issues in intensive care, which aims to evaluate risk factors for different adverse events, which includes infections.

AEs, defined as unintended injuries caused by health care management rather than the underlying condition of the patient [17], [18], currently represent one of the major challenges to improvement in quality of care. Their occurrence indicates the presence of important deficiencies in the process of care [19]. HAIs, an important category of AEs, have been studied for a longer time as an independent unit. Unfortunately, the research involving HAI is often not integrated with broader issues regarding other untoward events in healthcare.

At least 50% of the reported AEs were attributed to human errors. The Institute of Medicine report, *To Err is Human*, stated that preventable AEs in hospitalized patients represent the eighth leading cause of death in the United States, providing estimates of 44,000 to 98,000 deaths related to medical errors every year [20]. Some important facilitating conditions for the occurrence of error in healthcare have been described, with emphasis on patient age and severity on admission, presence of underlying diseases, hospitalization in University facilities, length of stay, presence of young and inexperienced HCW, lack of supervision, fragmentation of care, communication failures, insufficient nursing staffing and the related excessive workload [21]. It is noteworthy that the administrative dimension of healthcare contributes mostly to the occurrence of preventable adverse events when compared with patient-related conditions. Probably most of these unfavorable organizational general conditions may also contribute to the development of HAIs. It was our objective in this study to evaluate factors beyond those already considered “classical” risk factors for HAI.

Nurse staffing is always considered a potential factor leading to HAI [8]. Most studies have demonstrated an association but focus is mainly on absenteeism and evaluation of overtime is overlooked and multivariate analyses are seldom used [22]. Burnout of nursing staff has also been implicated as a risk factor for HAI [23]. When studying HAI, nurse staffing has been measured using nurse-to-patient ratios or nurse hours per patient-day [8] but, to our knowledge, this is the first study in which nursing workload was directly measured using a scoring system specifically designed for this.

Invasive devices are traditionally considered to be risk factors for infections and guidelines are directed to improve the care of these devices thus to minimize the risk of infection [1], [2]. On the other hand, the role of invasive devices in increasing the nursing workload is usually overlooked. The care required by invasive devices may represent an extra risk besides the risk posed by the device itself. Excessive nursing work load was significantly associated with the risk of acquiring HAI with an odds ratio of more than 11. Nursing workload was measured using NAS that is an evaluation of the proportion of nursing time required for each patient [15], adapted from the Therapeutic Intervention Scoring System (TISS) [24]. It has been observed that although it is used to evaluate nursing workload [25], the TISS does not take into account a number of nursing activities that are not directly related to the severity of the patients’ condition and that are not therefore evaluated, such as performing hygiene procedures, support of family members and administrative and managerial activities. The NAS includes the evaluation of such activities. After each working shift the HCW filled out an evaluation form for each patient under his/her care. The score represents the proportion of nursing time of one HCW necessary to care for that patient. Its value can surpass 100%, which means that for that patient more than one HCW is necessary for adequate nursing care. In our hospital, ICUs have one nursing HCW for 2 beds and in our step-down unit this proportion is of 3–4 beds per HCW. We decided to be conservative and to consider NAS≥51% as an excessive workload because patients moved between the ICUs and the step-down unit and it was not possible to estimate the amount of time spent in each unit. The higher proportion of non-compliance with the nurses’ patient care plans observed among the patients with HAIs may be intrinsically related to the detected excessive workload.

Besides nursing excessive workload, other important organizational issues were evaluated in our study, mainly those regarding communication failures, delays in performing multidisciplinary consultations, cancelation of medical invasive interventions and ward bed shortage. It is noteworthy that the majority of these occurrences could only be identified with the direct observation of physician and nursing rounds, since just a very small proportion of them were actually registered in the medical charts. Although organizational deficiencies are considered the main causes of errors [26], previous studies pointed out that caregivers are not used to registering organization problems in medical charts [27], [28]. Moreover, these administrative issues prolonged ICU stay unnecessarily among our patients and probably increased costs, and may have also contributed to the occurrence of HAIs.

Although this was not the object of our study and a multivariate analysis for prognostic factors was not done, in addition to suffering significantly more adverse events, the patients who developed HAI had a higher risk of dying during their hospital stay. This should be further analyzed.

This study has important limitations. It involved mainly medical ICUs, consequently, our findings cannot be generalized to all patients undergoing critical care, since surgical patients undergoing critical care were not included in the present study. On one hand, many surgical patients in intensive care are less complex when compared to medical patients. On the other hand, several studies show that adverse events related to surgery were the major cause of harm to patients in hospitals [29], accounting for 24.3 to 47.7% of events. In addition, our hospital provides care to highly complex patients, and although we observed both nursing and physician rounds daily, besides performing a detailed medical chart review, probably not all AEs were captured by our research team. Regarding nursing workload, we analyzed the average NAS related to the entire follow-up period. Although we had calculated the daily NAS for each patient, we did not analyze the excessive workload of each individual nurse considering only the two patients carried out by this nurse. The physician workload was not evaluated but, we believe that this was not an issue in our hospital because there are residents, fellows and attending physicians present in all shifts. However, a system devised to evaluate physician workload would be an interesting development. Finally, although we tried to control for variables previously associated with the occurrence of AEs and hospital mortality, other unknown confounding factors may have slipped our notice.

### Conclusions

In conclusion, excessive workload was the most important risk factor for HAI when evaluated together with other invasive devices. To our knowledge this prospective study is the first to quantify nursing workload using NAS in order to evaluate it as a potential risk factor for HAI.
